# Research article expression of surfactant protein-A and D, and CD9 in lungs of 1 and 30 day old foals

**DOI:** 10.1186/s12917-021-02943-5

**Published:** 2021-07-05

**Authors:** Tara Bocking, Laura Johnson, Amitoj Singh, Atul Desai, Gurpreet Kaur Aulakh, Baljit Singh

**Affiliations:** 1 Department of Veterinary Biomedical Sciences, Western College of Veterinary Medicine, University of Saskatchewan, Saskatoon, Canada; 2 Department of Small Animal Clinical Sciences, Western College of Veterinary Medicine, University of Saskatchewan, Saskatoon, Canada

**Keywords:** Tetraspanin, SP-A, SP-D, CD9, Horse

## Abstract

**Background:**

Respiratory diseases are a major cause of morbidity and mortality in the horses of all ages including foals. There is limited understanding of the expression of immune molecules such as tetraspanins and surfactant proteins (SP) and the regulation of the immune responses in the lungs of the foals. Therefore, the expression of CD9, SP-A and SP-D in foal lungs was examined.

**Results:**

Lungs from one day old (*n* = 6) and 30 days old (*n* = 5) foals were examined for the expression of CD9, SP-A, and SP-D with immunohistology and Western blots. Western blot data showed significant increase in the amount of CD9 protein (*p = 0.0397)* but not of SP-A and SP-D at 30 days of age compared to one day. Immunohistology detected CD9 in the alveolar septa and vascular endothelium but not the bronchiolar epithelium in the lungs of the foals in both age groups. SP-A and SP-D expression was localized throughout the alveolar septa including type II alveolar epithelial cells and the vascular endothelium of the lungs in all the foals. Compared to one day old foals, the expression of SP-A and SP-D appeared to be increased in the bronchiolar epithelium of 30 day old foals. Pulmonary intravascular macrophages were also positive for SP-A and SP-D in 30 days old foals and these cells are not developed in the day old foals.

**Conclusions:**

This is the first data on the expression of CD9, SP-A and SP-D in the lungs of foals.

**Supplementary Information:**

The online version contains supplementary material available at 10.1186/s12917-021-02943-5.

## Background

Horses are susceptible to many respiratory diseases which cause major economic and personal losses to the horse industry [1]. Diseases such as endotoxin-induced lung injury, recurrent airway disease and infectious diseases such as *Rhodococcus equi* cause lung inflammation which underlies clinical signs of respiratory diseases [2]. The role of various cells such as macrophages and neutrophils and immune molecules such as Toll-like receptors is under active investigation [2, 3]. The advances made so far have created a better understanding of the mechanisms of respiratory diseases of the horse; however, much remains to be revealed.

Lung surfactant is composed of a complex mixture of lipids and proteins [4]. Surfactant deficiency may occur in premature foals and is a leading cause of respiratory distress syndrome [5]. Surfactant also functions in pulmonary host defence as a barrier to pathogens and for enhancing muco-ciliary transport [4].There are four surfactant proteins named SP-A, SP-B, SP-C and SP-D. Along with phospholipids, SP-B and SP-C play a role in preventing alveolar collapse by helping to reduce surface tension and assisting with redistribution and production of surfactant. SP-A and SP-D are larger glycoproteins of 26-36 kDa and 43 kDa, respectively [6]. These proteins are hydrophilic and have significant immunoregulatory functions in infectious diseases of the lung [7]. SP-A and SP-D are members of the collectin family of proteins and have shared and distinct functions in their carbohydrate-binding abilities and interactions with pathogens. Collectin proteins have been shown to opsonize both gram-positive and gram-negative bacteria, viruses, allergens and fungi [8].

 Horses are uniquely sensitive to endotoxic shock from gram-negative bacterial infections [9]. Endotoxins are lipopolysaccharides released during gram negative bacterial cell death [10]. SP-A and SP-D have been shown to interact with gram-negative bacteria such as *Escherichia coli* and *Pseudomonas aeruginosa* [11]. Both proteins bind to LPS and facilitate bacterial clearance through different components and mechanisms [4]. The potential role of serum SP-D in diagnosing inflammatory airway disease in horses has been recently reported [12]. Considering the potential role of SP-A and SP-D in respiratory biology of the horse, there is a need for more information on their expression in these species.

CD9 is a 24-27 kDa cell-signalling surface molecule belonging to the tetraspanin family of proteins which are expressed on a number of both normal and malignant cell types [13]. CD9 spans the cell membrane four times allowing it to easily associate with other membrane proteins, integrins, growth factors and intracellular signalling molecules [14]. Functions of CD9 include cellular growth, development, activation, adhesion, and motility [13]. CD9 plays a role in fertilization, tumor metastasis and platelet activation. CD9 has been reported as a negative regulator of LPS induced lung inflammation [14] and the loss of CD9 was shown to increase macrophage activation and TNF-α production in mice in vivo. CD9 distribution has been previously studied in human, mouse and swine tissues. CD9 homologues in equine have been identified and these share significant homology with the human CD9 [15, 16]. There however are no data on the protein expression of CD9 in lungs of foals.

The purpose of the current study is to evaluate possible differences in the expression of the immunological proteins SP-A, SP-D and CD9 between healthy one day and 30 day old foals.

## Results

### CD9 Expression

**Fig. 1:**
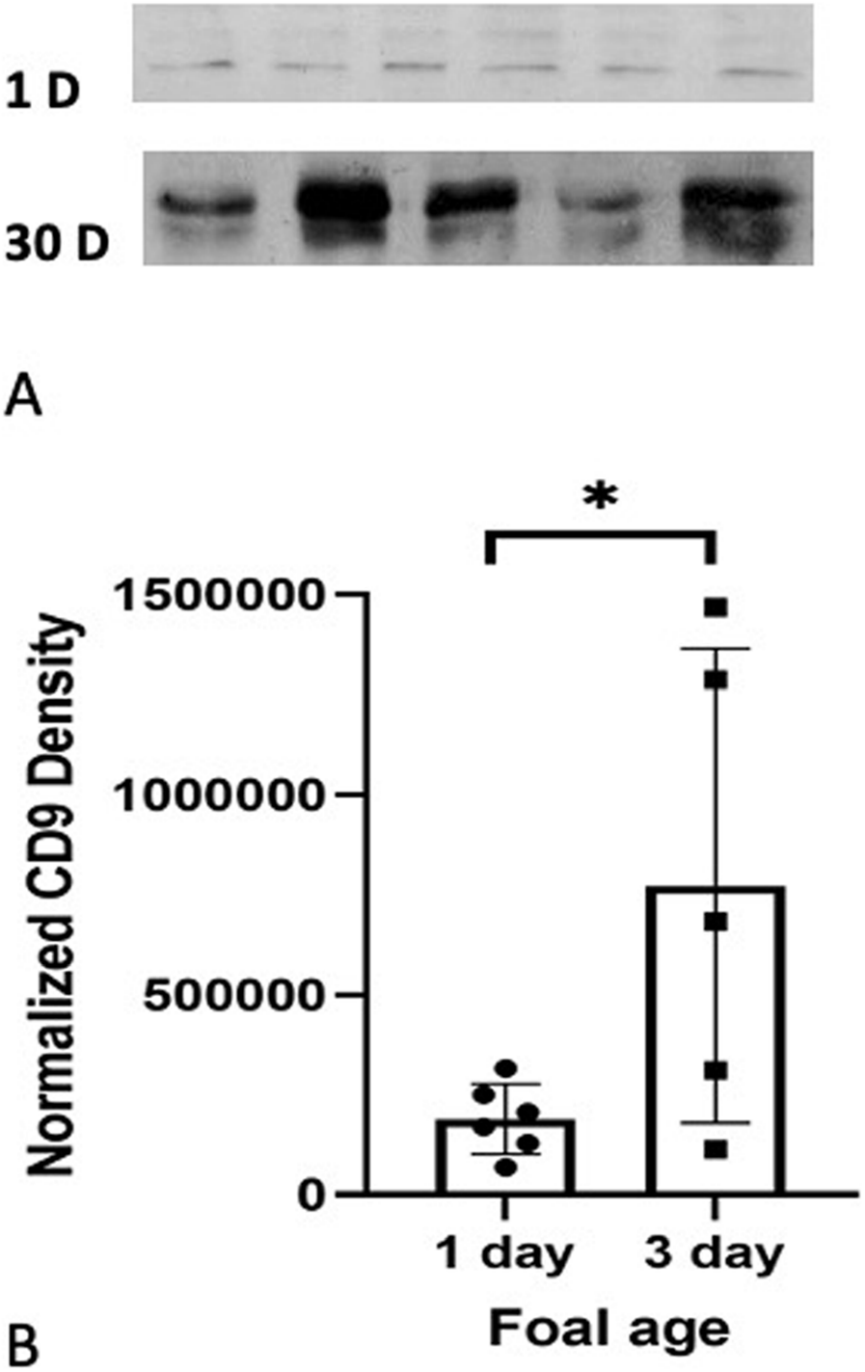
The representative western blots show 27 kDa CD9 protein in 1 day (1 D) (*N* = 6) and 30 days (30 D) (*N* = 5) old foal lung tissues (**A**). The blot quantification (**B**) from duplicates showed a significant increase in the amount of CD9 in lungs, as determined by normalized density (a.u.) of the CD-9 protein blot, of 30 day old foals (772,861 ± 264,872 a.u.) compared to the one day foals (190,430 ± 35,927 a.u.); * *p* = 0.0397. The full-length blots/gels are presented in Supplementary Fig. 1

**Fig. 2:**
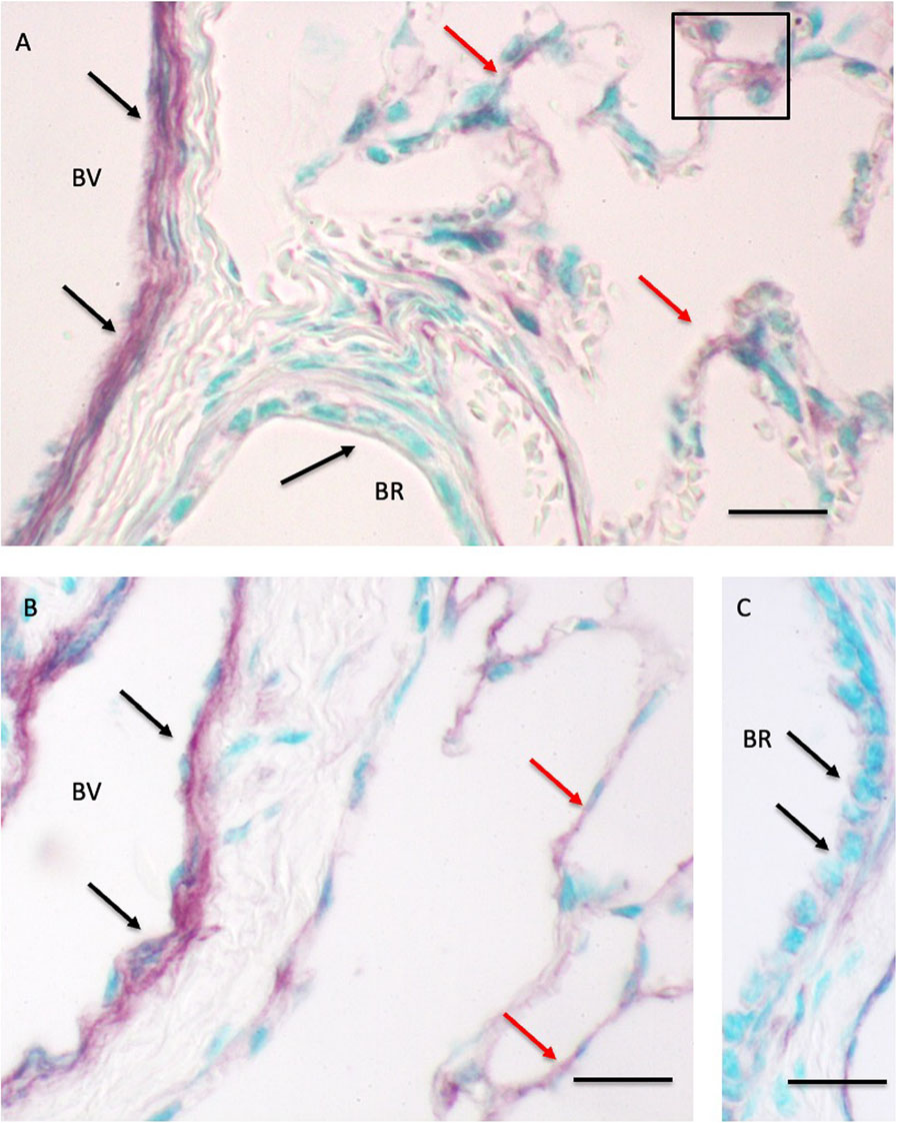
Immunohistology for CD9 protein: The CD9 staining is observed in the vascular endothelium (black arrows) of blood vessels (BV) and alveolar septa (red arrows) of one day old (**A**) and 30 day old (**B** and **C**) foals. The bronchiolar (BR) epithelium is negative (black arrows). Bar = 20 μm

Western blots showed bands at approximately at 27 kDa for CD9 (Fig. 1). The quantification of blots showed a 4.1 fold increase (*p = 0.0397*) in the amount of CD9 protein in lung homogenates of 30 day old foals (772,861 ± 264,872 a.u.) compared to one day (190,430 ± 35,927 a.u.) old foals (Fig. 1). CD9 staining was weak in the alveolar septa, absent in bronchiolar epithelium and present in the endothelium of large blood vessels of both one day (Fig. 2 A) as well as 30 day old foals (Fig. 2B C, Table 1).

**Table 1:** Staining intensity comparison of the immunohistochemical distribution of CD9 in equine lung tissues between groups. Level of staining was scored from + (weakly positive) to +++ (strongly positive)

Lung	CD9		SP-A		SP-D	
	*One Day Foals*	*30 Day Foals*	*One Day Foals*	*30 Day Foals*	*One Day Foals*	*30 Day Foals*
Alveolar septa	+	+	+++	++	++	+++
Smooth muscle	-/+	-/+	-/+	-/+	-/+	-/+
Bronchiolar Epithelium	-	-	++	+++	+	++
Vascular Endothelium	+	+	+++	++	+++	+++
Macrophages	+	+	+	+	+	++

### SP-A expression

**Fig. 3:**
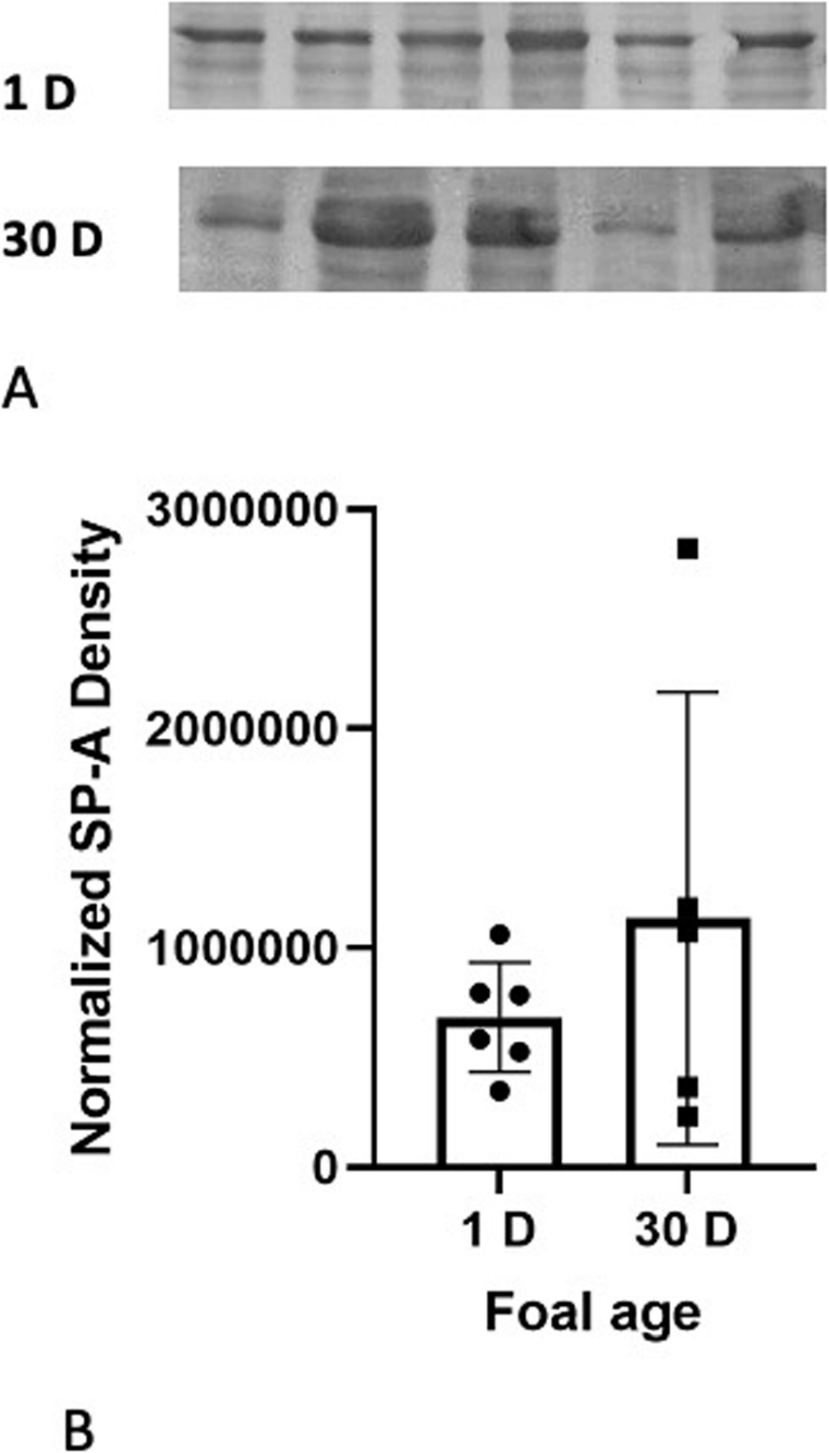
The representative western blot analyses of 35 kDa SP-A proteins in 1 day (1 D) and 30 days (30 D) old foal lung tissues (**A**). The blot quantification (**B**) from duplicates showed no difference in the amount of SP-A in lungs, as determined by normalized density (a.u.) of the SP-A protein blot, of 30 day old foals compared to the one day old foals

**Fig. 4:**
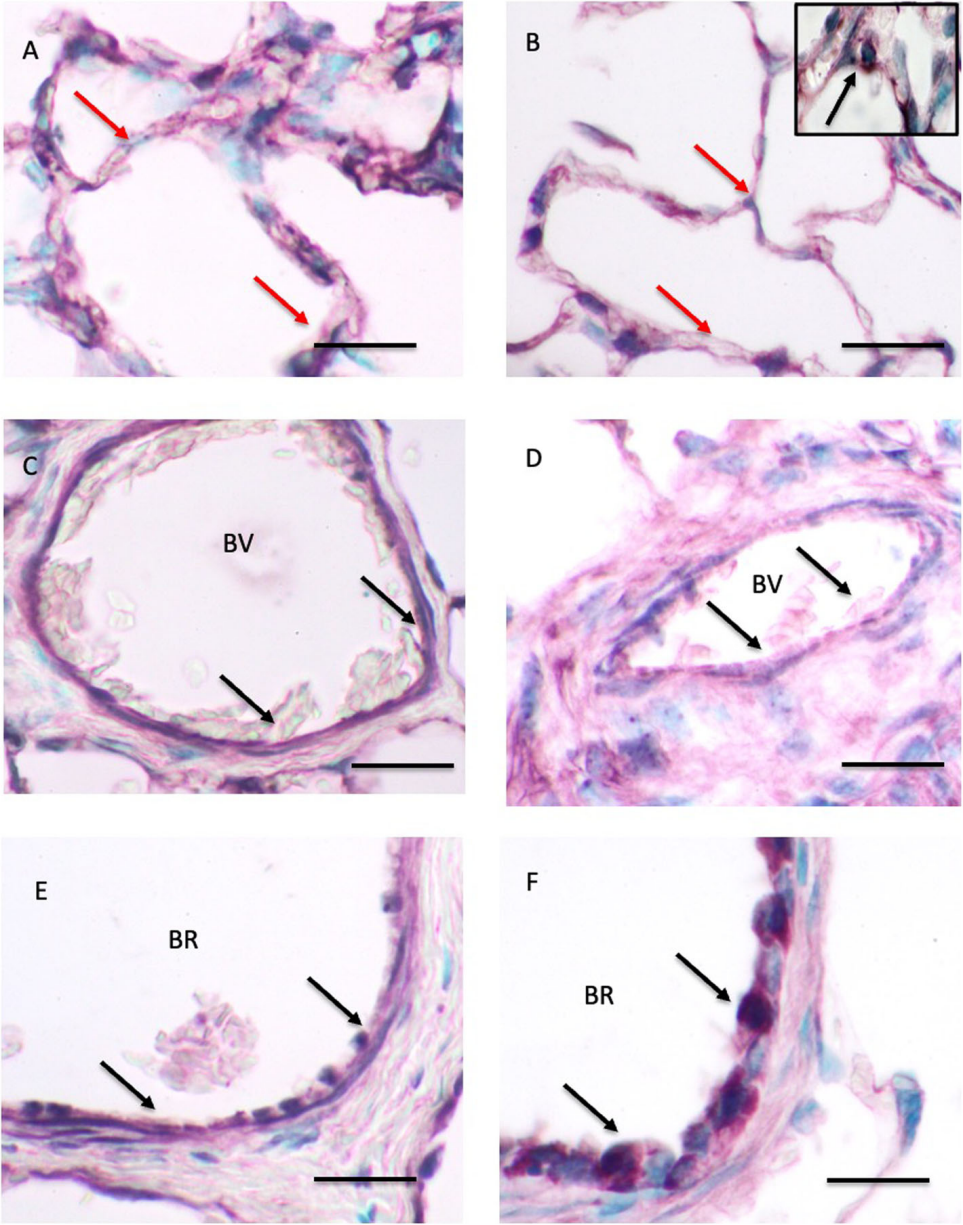
Immunohistology for SP-A protein: The SP-A staining is observed in the alveolar septa (read arrows), vascular endothelium (black arrows) of blood vessels (BV; black arrows), and bronchiolar epithelium (BR; black arrows) of one day old (**A**, **C**, **E**) and 30 day old (**B**, **D**, **F**) foals. The inset in **B** shows staining (arrow) in alveolar type II epithelial cell. Bar = 20 μm

SP-A expression The analysis detected bands at approximately 35 kDa for SP-A which are consistent within the range of expected sizes for all three proteins. The quantification revealed no differences in the amounts of SP-A proteins in lung homogenates from one day (681,148 ± 102,496 a.u.) and 30 day (1,133,761 ± 461,402 a.u.) old foals (Fig. 3). SP-A staining was observed in alveolar septa of 1 day (Fig. 4 A) and 30 day old (Fig. 4B) foals. The staining was localized in the type II alveolar epithelial cells (Fig. 4B inset). The vascular endothelium in the lungs of one day old (Fig. 4 C) and 30 day old (Fig. 4D) foals was positive for SP-A protein. The SP-A staining was prominent in the bronchiolar epithelium in the lungs of both the age groups (Fig. 4E F).

### SP-D expression

**Fig. 5:**
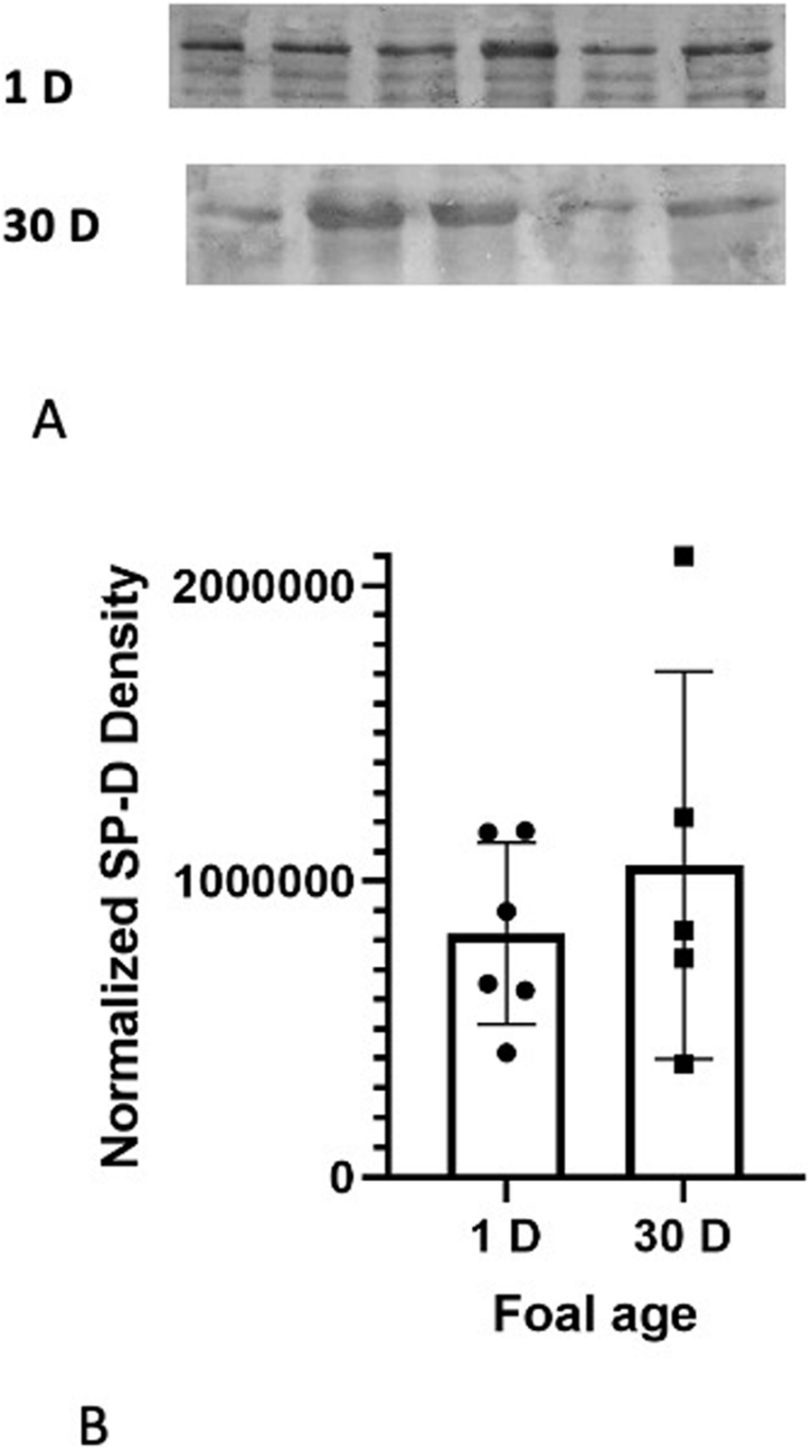
The representative western blot analyses of 43 kDa SP-D proteins in 1 day (1 D) and 30 days (30 D) old foal lung tissues (**A**). The blot quantification (**B**) from duplicates showed no difference in the amount of SP-D, as determined by normalized density (a.u.) of the SP-D protein blot, in lungs of 30 day old foals compared to the one day old foals

**Fig. 6:**
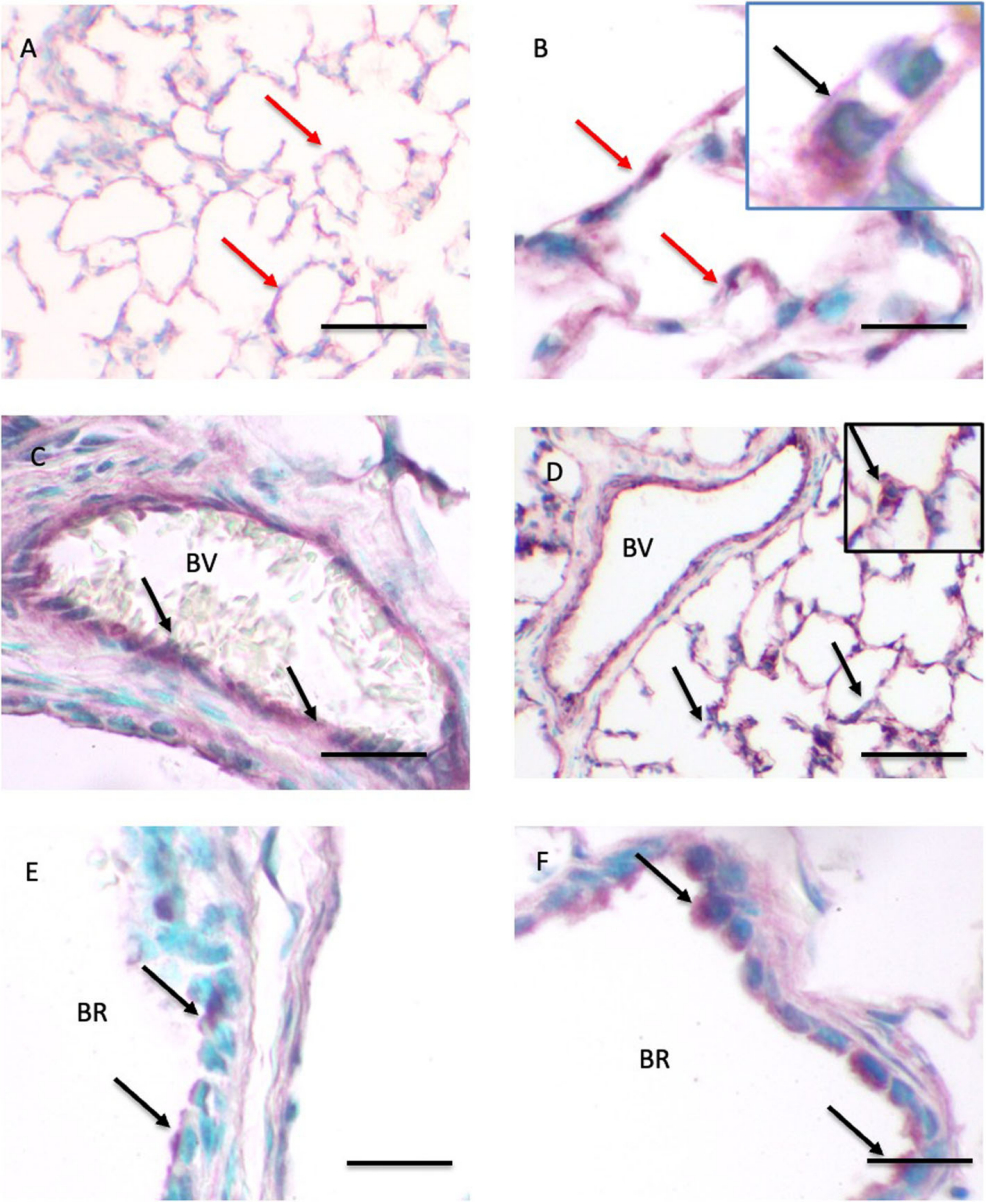
Immunohistology for SP-D protein: The SP-D staining is observed in the alveolar septa (red arrows), vascular endothelium (arrows) of blood vessels (BV; black arrows), and bronchiolar epithelium (BR; black arrows) of one day old (**A**, **C**, **E**) and 30 days old (**B**, **D**, **F**) foals. The inset in **B** shows staining (black arrow) in a pulmonary intravascular macrophage and the inset in D shows staining ( black arrow) in type II alveolar epithelial cell. Bar = 100 μm (**A**, **C**, **D**) Bar = 20 μm (**B**, **E**, **F**)

The analysis detected bands at approximately 43 kDa for SP-D which is consistent within the range of expected sizes for all three proteins (Fig. 5). The quantification revealed no differences in the amounts of SP-D proteins in lung homogenates from one day (822,514 ± 125,215 a.u.) and 30 day (1,053,623 ± 292,947 a.u.) old foals (Fig. 5). The immunohistochemistry showed diffused SP-D staining in the alveolar septa in lungs of one day old foals (Fig. 6 A). The septal staining in the lungs of 30 day old foals was more discrete (Fig. 6B C) and appeared to be localized into septal macrophages/monocytes (Fig. 6B inset). Similar to SP-A, there was staining for SP-D in the endothelium of large blood vessels in lungs of foals of both the age groups (Fig. 6 C, 6D). Bronchiolar epithelium in lungs of one day old foals (Fig. 6E) showed lower staining compared to that in the 30 day old foals (Fig. 6 F).

## Discussion

This paper contains one of the first data on the expression of SP-A, SP-D and CD9 in the lungs of neonate and 30 day old foals. The data obtained with western blots and immunohistology show significant increase for CD9 but not for SP-A and SP-D expression in 30 day old compared to one day old foal.

There are very limited in situ CD9 protein expression data in the lung. Most of the data are related to lung cancer [17] and in this context out data is significant. Our data show expression of CD9 in vascular endothelium and alveolar septa in lungs from one day and 30 day old foals with a significant increase in 30 day old foals. Interestingly, the experiments did not detect CD9 expression in bronchiolar epithelium of either of the age groups. Considering the role of CD9 in cell proliferation [17], the increase in expression may be related to the ongoing expansion of the lung parenchyma as reported by us previously through rigorous morphometric evaluation [18]. Tetraspanin has many roles ranging from cell motility and proliferation in many cell types including endothelial cells [19]. This protein can lead to activation of platelets through formation of complexes with integrin αvβ3 [20]. Therefore, the increased expression of CD9 over the first month of life of foals may suggest a maturing of vascular physiological and immunological capacity of the lung. The increased expression of CD9 may be critical in dampening inflammatory responses in the lungs of neonate foals as CD9 negatively regulates lung inflammation in mice by reducing localization of TLR and CD14 in lipid rafts [14]. The anti-inflammatory role of CD9 also may fit with our previous observations on the reduced localization of TLR2 in lipid rafts of neonate foals [21]. Taken together, the increased expression of CD9 may protect the lungs of neonate foals against excessive inflammation.

Surfactant is comprised of phospholipid and protein components. The surfactant proteins (SP) are produced by alveolar type II epithelial cells as well as non-ciliated bronchiolar epithelial cells (Clara cells) and are important in pulmonary physiology and immunology. [22, 23]. These are the first data on the expression of SP-A and SP-D in lungs of foals. In contrast to CD-9, the western blots revealed no change in the expression of SP-A and SP-D between one day old and 30 day old foals. The immunohistology showed robust localization of both SPs in alveolar septa, airway epithelium and vascular endothelium. As expected, both proteins were localized in type II alveolar epithelial cells and its diffused expression along the septa is due to the spread of the surfactant along the alveolar epithelium. The intensity of SP-D expression in the bronchiolar epithelium which may likely be an outcome of increase in the size and number of cells. The day old foals lack pulmonary intravascular macrophages (PIMs) as these develop over the first four weeks of age [18]. SP-D was clearly noticed in the PIMs in 30 day old foal. Previous studies have mostly detected the expression of SPs in the airway epithelial cells and macrophages [24]. Interestingly, the vascular localization of SP-A and SP-D has been only occasionally reported mostly in diseased pulmonary and non-pulmonary tissues [24–26]. The role of the vascular SP may be important considering the recent finding that serum SP-D may be of diagnostic value in inflammatory airway disease of the horse [12]. This also points us to study the expression of SP-A and SP-D in diseases such as *R. equi* infection in the foals. Our data showing robust staining of pulmonary vascular endothelium is thus intriguing. It is possible that the staining is an outcome of circulating SP-A and SP-D attached to the endothelial surface. Another possibility could be translocation of alveolar SP-A and SP-D across the delicate alveolar septa of neonate foals into the pulmonary vascular compartment. The presence of SP-A and SP-D in the pulmonary vascular compartment may provide the foal lung with another mechanism to fight blood-borne pathogens.

Taken together, the data included in this manuscript show baseline expression of SP-A, SP-D and CD9 in foals and the changes in the expression between one day and 30 day old foals. Considering the role of these proteins in lung defense, the new data set the stage for further studies.

## Methods

### Animal groups

 The experimental protocols for this study were approved by the Animal Care Assurance Committee of the University of Saskatchewan in accordance with the national guidelines for the Canadian Council of Animal Care. The samples used in these experiments were collected previously and required no new animals thus reducing the number of animals used in experimental studies [18]. Eleven foals were born to mixed breed draft-type mares housed at the College’s Animal Care Facility. The selection criteria for the selection of foals was decided before the start of the experiments. It included that foal should be born within the same foaling season, be found healthy upon a full physical examination by a veterinarian to ensure maturity and healthy airways, and show no sign of inflammation based on blood examination. We decided to allocate the first five foals born to the 30 day old group (n = 5) and the remainder were assigned to the one day old group (*n* = 6). The sample size was limited by the numbers of foals available and no *a priori* sample size calculation was performed. These foals were euthanized with of pentobarbital sodium (75-100 mg/kg) and have previously been described [18].

### Tissue collection and processing

The foals were euthanized and tissues were collected. Foal lungs were fixed *in situ* by pouring 0.1 % glutaraldehyde, phosphate buffered at pH 7.4 and 4 % paraformaldehyde, pH 7.2–7.4 into the left bronchus. Fixative was poured until flow ceased, approximately 1 L for 1 day old foals and 2 L for 30 day old foals. The entire left lung was removed and stored in a sealable container of fixative for 24 h at 4^0^ C. Tissues were embedded in paraffin for immunohistochemistry analysis and the right lung was snap frozen and stored at -80^0^ C for Western blot analysis.

### Immunohistochemistry for SP-A, SP-D and CD9

Five µm sections were cut from paraffin blocks and placed on glass slides coated in Poly-L-lysine. The tissue sections were dewaxed, dehydrated and rehydrated. Slides were treated with H_2_O_2_ (0.5 %) in methanol for 20 min, pepsin (Sigma Life Sciences, St. Louis, MO) for 60 min and bovine serum albumin (1 %) (Sigma Life Sciences, St. Louis, MO) for 30 min. The slides were incubated overnight at 4^0^ C with exposure to commercially available rabbit anti-SP-A polyclonal antibody (1:50 − 1:500, Bioss Inc., Woburn, MA), rabbit anti-SP-D/SP-D polyclonal antibody (1:50 − 1:150, Bioss Inc., Woburn, MA) and mouse anti-CD9 (H19a) (1:25, Biolegend, Santa Cruz, CA) primary antibodies. Secondary antibodies (1:100; Polyclonal goat anti-mouse IgG and polyclonal goat anti-rabbit IgG, Dako Canada Inc., Burlington, ON) were applied to the tissues for 30 min. The CD9 (H19a) has been used in equine tissues previously [15]. The tissues were color developed with a commercial vector kit (Vector VIP Peroxidase Substrate Kit, Vector Laboratories Inc., Burlingame, CA) and counter stained with methyl green (Vector Laboratories Inc., Burlingame, CA). IHC controls included tissues with either anti-von Willebrand factor (Dako Canada Inc., Burlington, ON) as the primary antibody or primary antibody exclusion or isotype matched immuno-globulins.

The lung sections stained with CD-9, SP-A and SP-D antibodies were scored with a method adapted from a previously described scoring system and the evaluator was blinded to the identity of the groups [27]. Five fields from each tissue section were observed at 100X under oil immersion and were scored for overall staining intensity, proportion of positively stained type II cells, positively stained alveolar macrophages and vascular endothelium.

Tissue sections stained for CD9, SP-A and SP-D were assessed for reactivity in different areas of the lung by an evaluator who was blinded to the identity of the samples. Staining intensity was scored from + (weak staining), ++ (moderate staining) and +++ (strong staining).

### Microscopy

 Images were acquired under 100X high power field at Olympus microscope.

###  Western blotting

Snap frozen lung tissues (0.1 g) collected from the right lung of 1 day and 30 days old foals were homogenized in 2.5 mL Tissue Protein Extraction Reagent (T-PER) (Thermo Scientific, Rockford, IL) and centrifuged at 1,500 rpm for 16 min at 4˚C. The supernatant was collected and combined with 2X sodium dodecyl sulfate (SDS) sample buffer (Sigma Life Sciences, St. Louis, MO). Samples were boiled at 96˚C for 3 min to denature the protein. Samples were loaded onto 12 % polyacrylamide gel and transferred to a Hybond ECL blotting membrane (GE Healthcare). One lane was loaded with Novex Sharp pre-stained protein standard (Invitrogen, Carlsbad, CA) and a control lane was loaded with SDS sample buffer. The membrane was blocked for one hour in 5 % skim milk in 1XPBS-Tween20 and incubated overnight at 4˚C with primary antibody, rabbit anti-SP-A polyclonal antibody, unconjugated (1:50 − 1:500, Bioss Inc., Woburn, MA), rabbit anti-SP-D/SP-D polyclonal antibody, unconjugated (1:50 − 1:150, Bioss Inc., Woburn, MA) and mouse anti-CD9 (H19a) (1:25, Santa Cruz Biotechnology Inc., Santa Cruz, CA). All primary antibodies were diluted in 5 % skim milk in 1XPBS-Tween20 solution. The membrane was washed twice in 1XPBS-Tween20 for 15 min each time, incubated for 1 hour at room temperature in secondary antibody (Polyclonal goat anti-mouse IgG and polyclonal goat anti-rabbit IgG, Dako Canada Inc., Burlington, ON) and washed again four times in 1XPBSTween20. Secondary antibodies were diluted in 1XPBS-Tween20. Detection was performed using 3, 3’-diaminobenzidine (DAB) (Abcam, Cambridge, MA) in the presence of H_2_O_2_.

A uniform rectangular lane area, or the region of interest (ROI), was drawn for each sample lane (*N* = 6 for 1 day foals, *N* = 5 for 30 day foals), by the open image quantification software, Image J-Fiji, to measure the total pixel counts and intensity (i.e. integrated density expressed as arbitrary units (a.u.)) in each lane, before and after image inversion. Thus, the pixel intensity was normalized for local background in each ROI. This is referred to as the normalized integrated density (a.u.) i.e. corrected for background signal for a sample lane. Fold differences in average normalized pixel density were computed for 1 and 30 day foal lung homogenates and finally, the data was analyzed by unpaired two-tailed t-test for testing the differences between 1 and 30 day foals. All 8-bit images were uniformly adjusted for local contrast, for visualization purpose only.

### Statistical analysis

Western blot normalized integrated density for the proteins was expressed as mean ± S.D. (a.u.). Unpaired student’s t-test was used to analyze the differences between 1 and 30 day old foal protein expression (data followed normal distribution), as determined by the western blot analysis. Data were analyzed using Graphpad Prism software, LLC (v9.0).

## Supplementary Information

**Additional file 1.**

## Data Availability

The datasets used and/or analysed during the current study available from the corresponding author on reasonable request.

## Abbreviations

SP: Surfactant Protein
TLR: Toll-like Receptor
