# Supplementary material for: Research article expression of surfactant protein-A and D, and CD9 in lungs of 1 and 30 day old foals
Source: BMC Vet Res. 2021 Jul 5;17:236. doi: 10.1186/s12917-021-02943-5 (PMC8256609; doi:10.1186/s12917-021-02943-5)

**Supplementary Data**

**Expression of Surfactant Protein-A and D, and CD9 in Lungs of 1 and 30 Day Old Foals**

^1^Tara Bocking, ^1^Laura Johnson, ^1^Amitoj Singh, ^1^Atul Desai, ^2^Gurpreet Kaur Aulakh and ^1^Baljit Singh

Department of ^1^Veterinary Biomedical Sciences, ^2^Small Animal Clinical and Sciences, Western College of Veterinary Medicine, University of Saskatchewan, Saskatoon, Canada

**Correspondence:**

Baljit Singh, FCAHS, BVSc&AH, PhD

Email: [baljit.singh@usask.ca](mailto:baljit.singh@usask.ca)

**Supplementary Figures:**

**Supplementary Fig. 1:** The full-length blots/gels show specific protein bands marked by arrows for CD9, SP-A and SP-D protein in 1 day (1 D) (N=6; F1-F6) and 30 days (30 D) (N=5; F1-F5) old foal lung tissues. Ladder is annotated as L and the specific protein bands corresponding to the upper (260 kDa) and lower range (3.5 kDa) M.W. are labelled for reference.


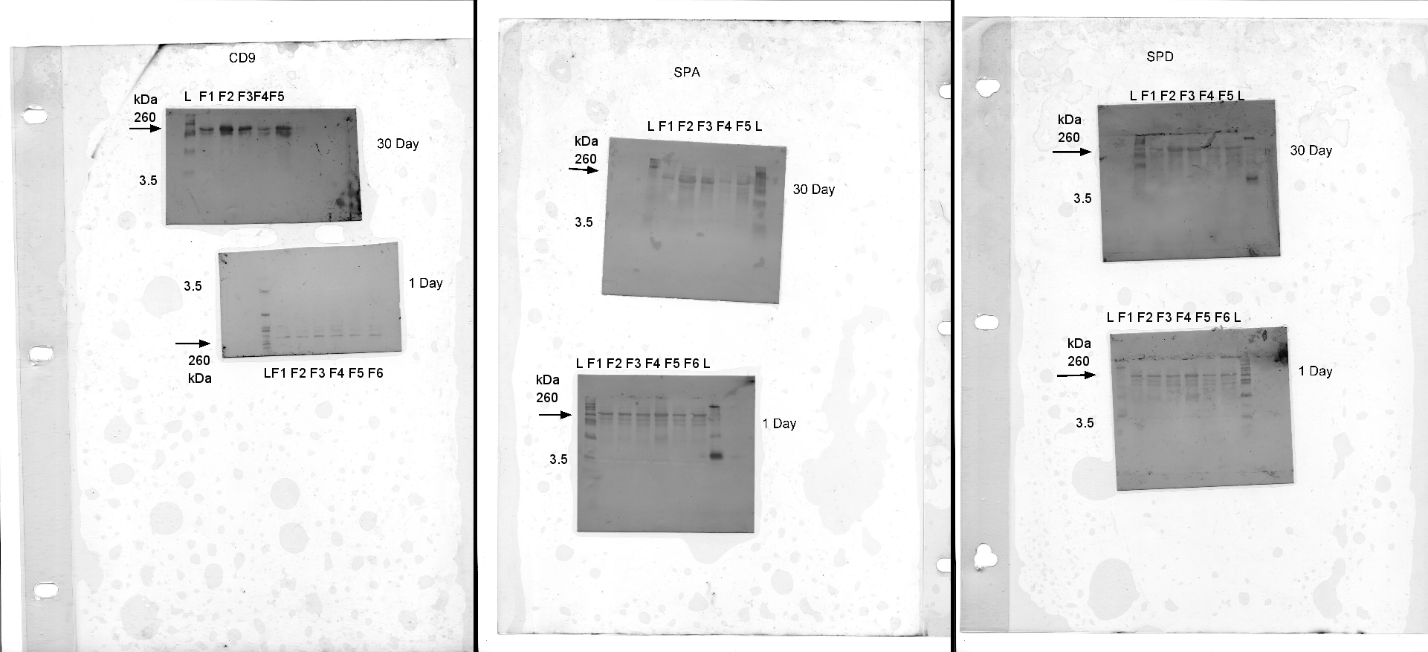

Supplement: Supplementary file 1 — Additional file 1. [file 12917_2021_2943_MOESM1_ESM.docx]
